# Supplementary figures and images for: Diversity in the internal functional feeding elements of sympatric morphs of Arctic charr (Salvelinus alpinus)
Source: PLoS One. 2024 May 21;19(5):e0300359. doi: 10.1371/journal.pone.0300359 (PMC11108142; doi:10.1371/journal.pone.0300359)

(A)

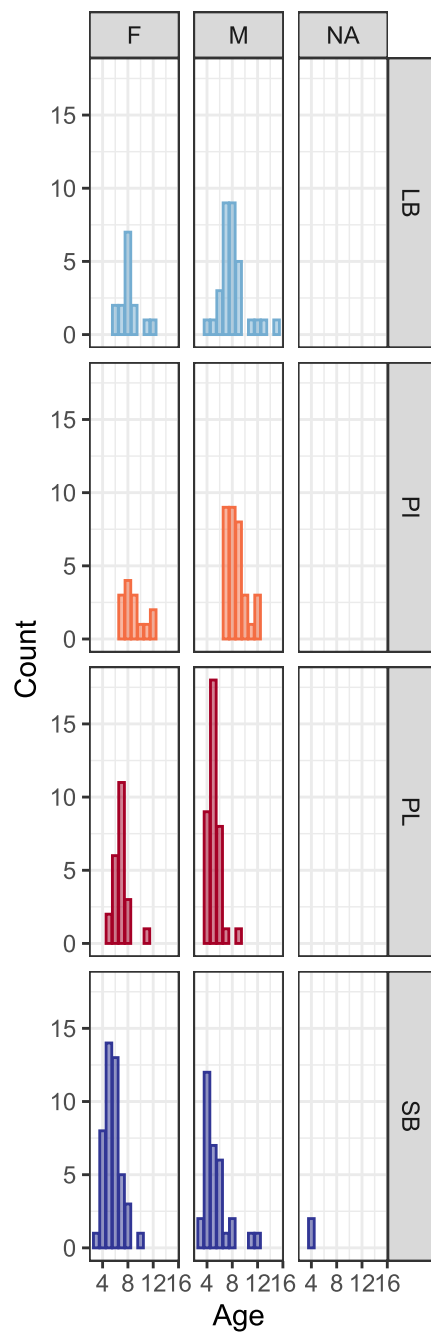

(B)

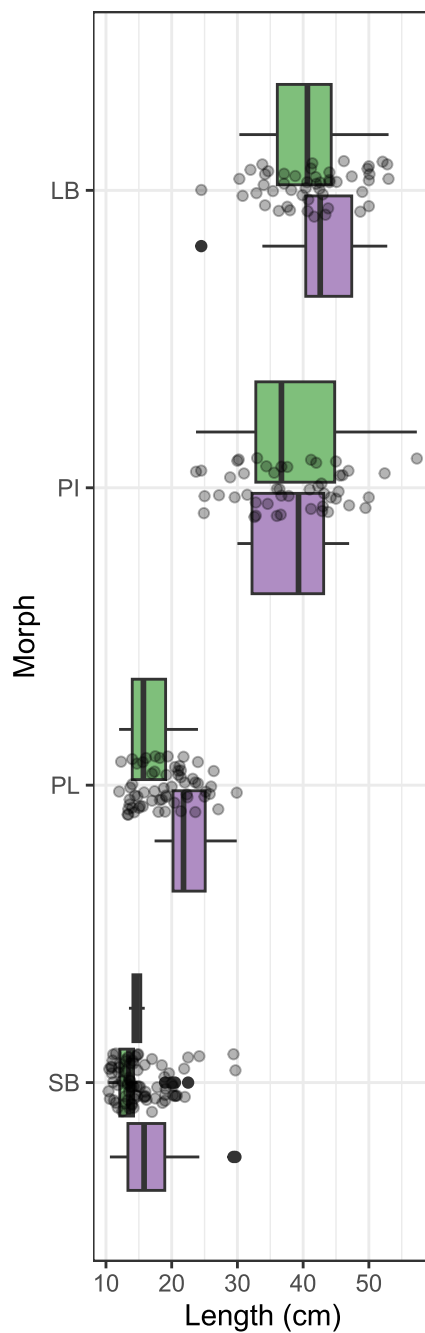

(C)

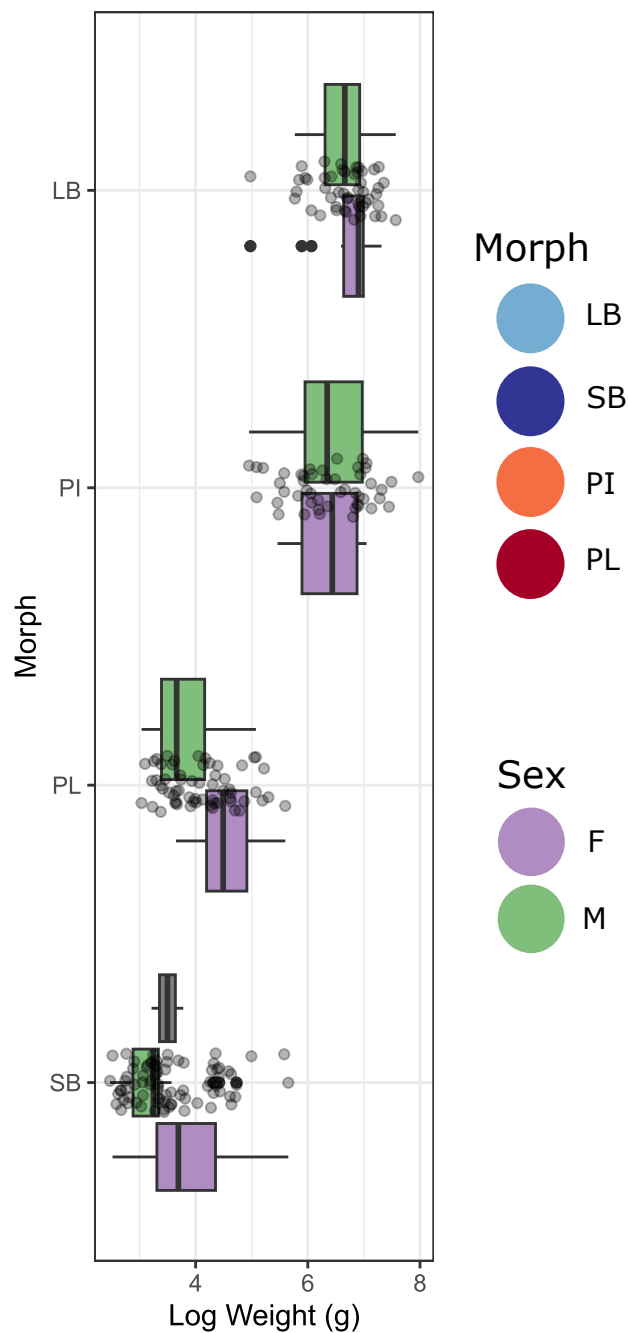

Morph

LB

SB

PI

PL

Sex

F

M

Supplement: S4 Appendix — (A) Histogram, showing age (years) distribution for all morphs by sex (NA indicates SB that could not be sexed). (B) The variation in fork length (FL, cm) by morph by sex represented by a boxplot. (C) The variation in loge weight (g) by morph by sex are represented in boxplots. (PDF) [file pone.0300359.s004.pdf]

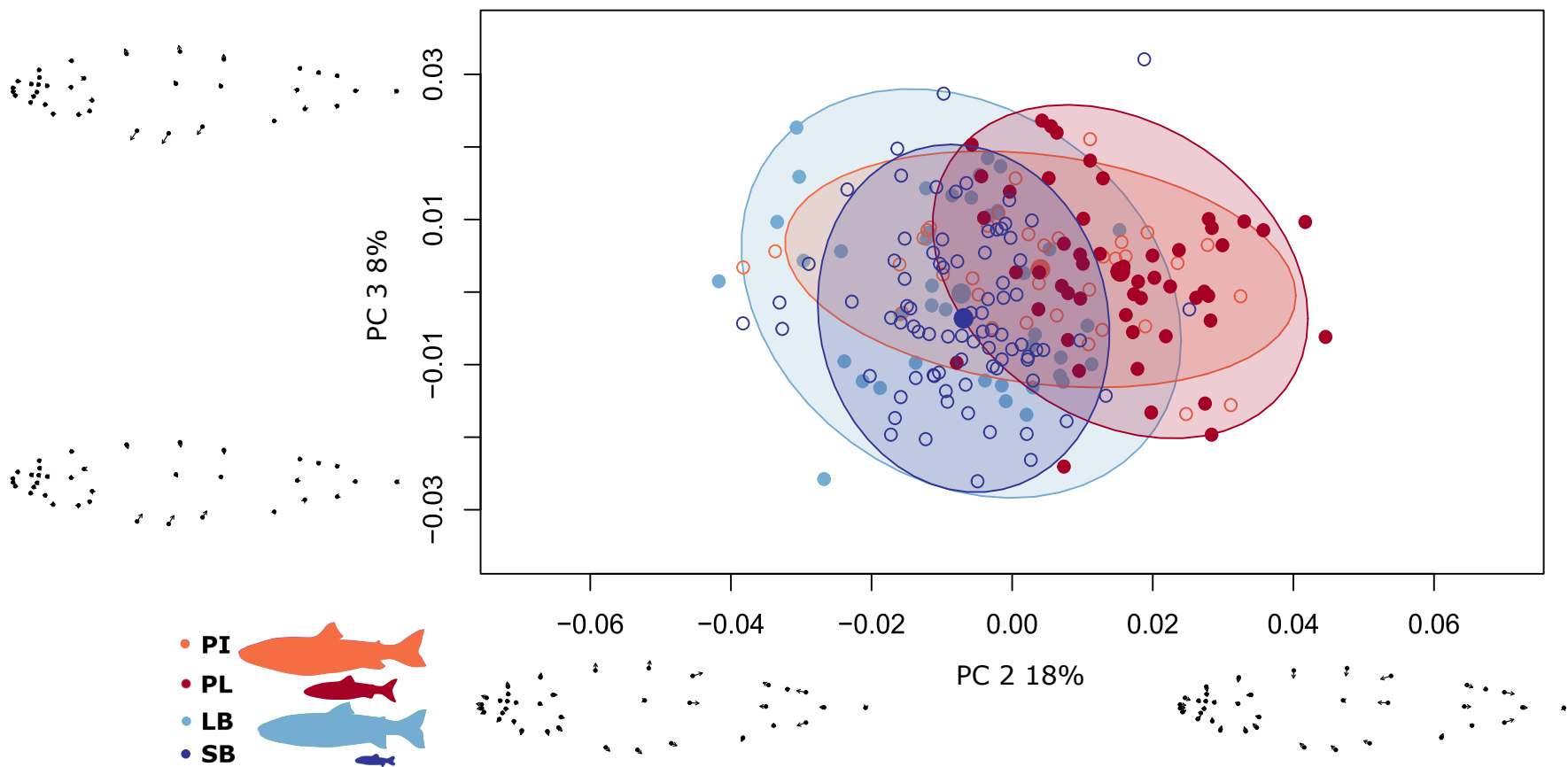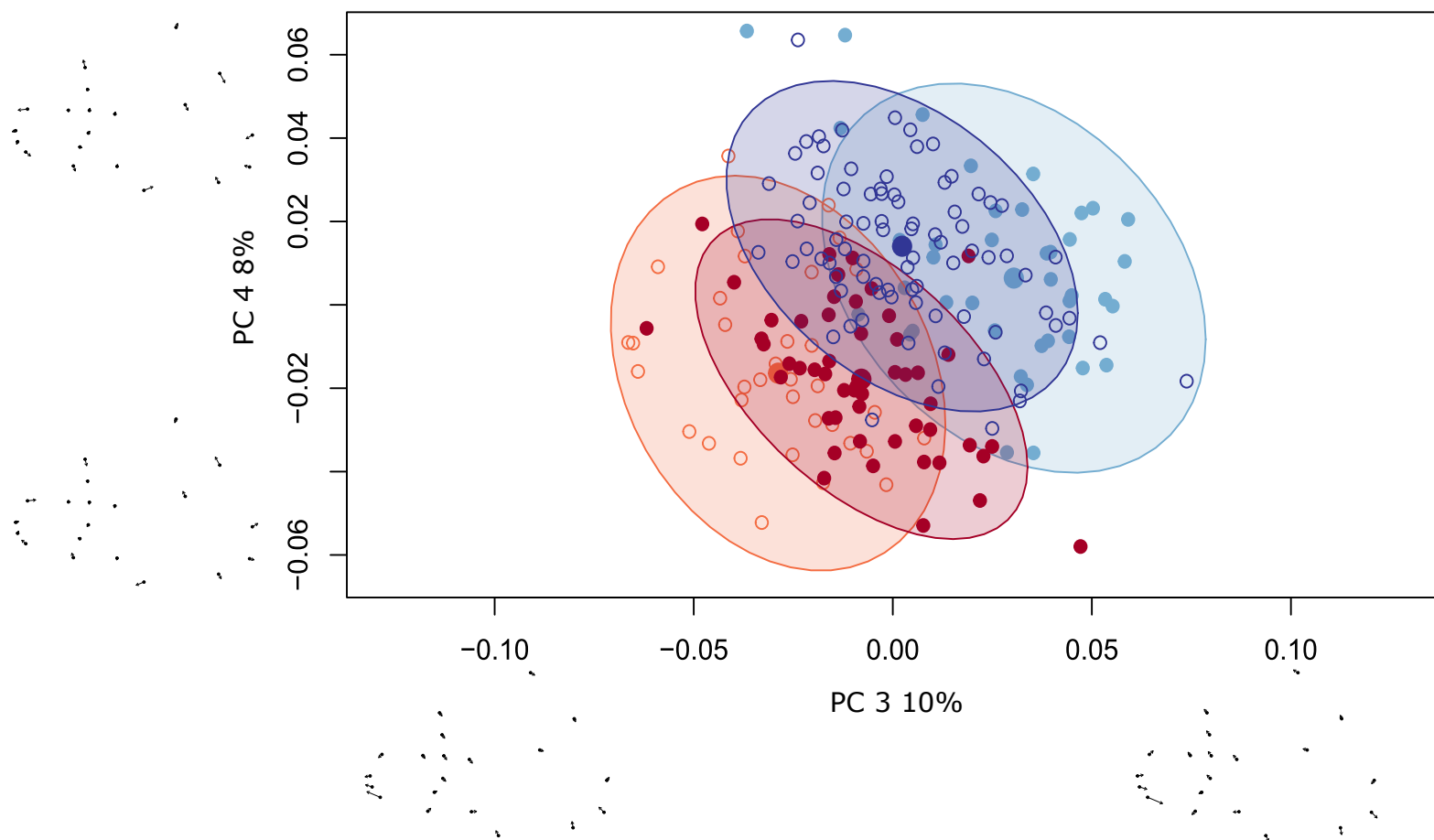

Supplement: S7 Appendix — Plots of shape warps on X- and Y-axis are unmagnified. Each dot represents an individual and the ellipses represent 95% CI for the distribution by morph (large dot represents the mean of each morph distribution in these two dimensions of shape). For the whole-body (top) PC2 and 3 explain 18% and 8% of the variation respectively (PC1 was biased by sampling error, and not depicted) and for the head shape (bottom) PC3 and 4 explain 40% and 22% respectively (PC1 and PC2 were biased by sampling error, and not depicted). (PDF) [file pone.0300359.s007.pdf]

Trials

● rep\_1

● rep\_2

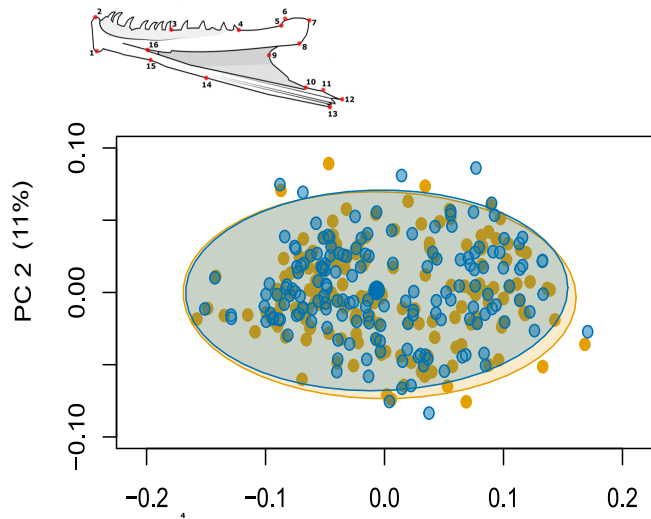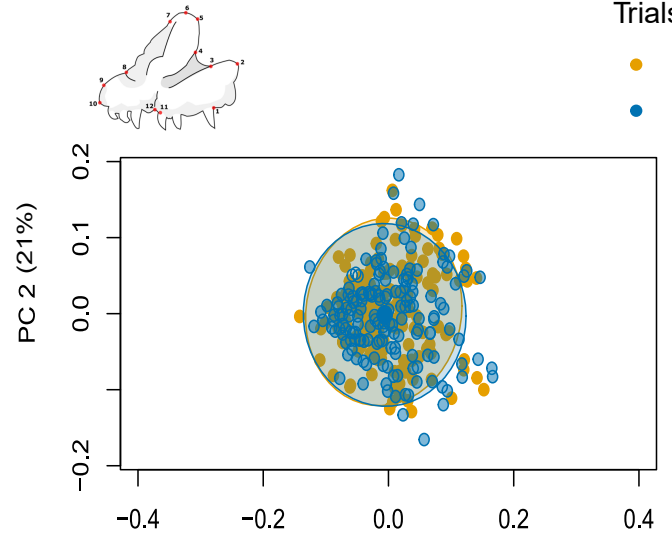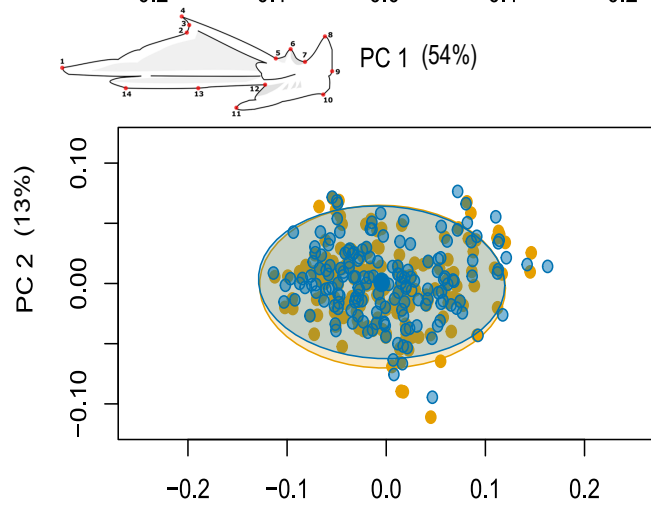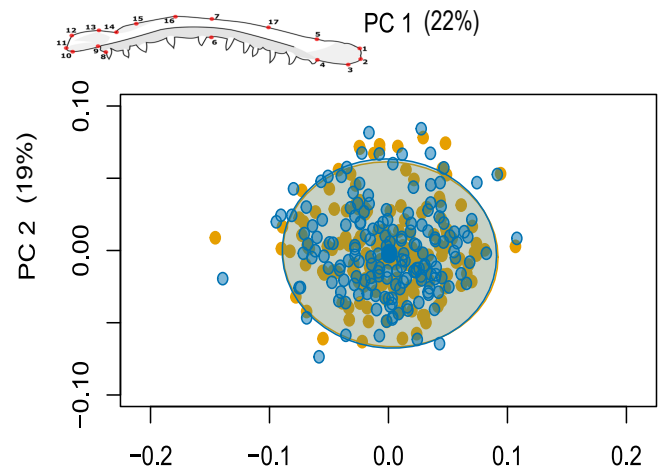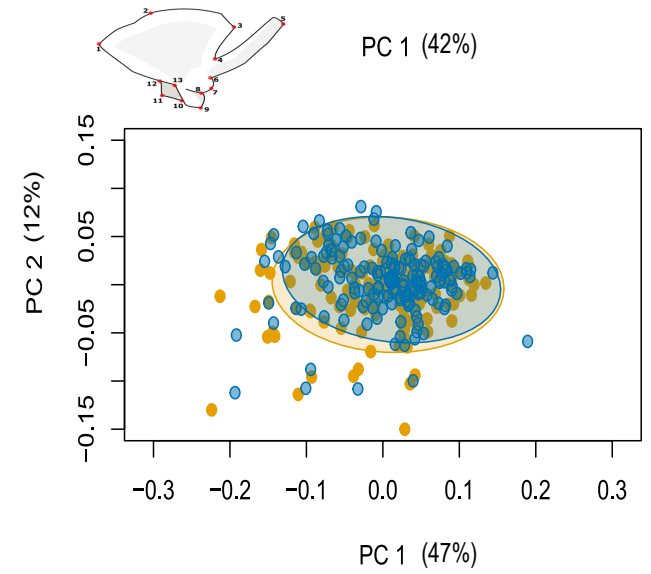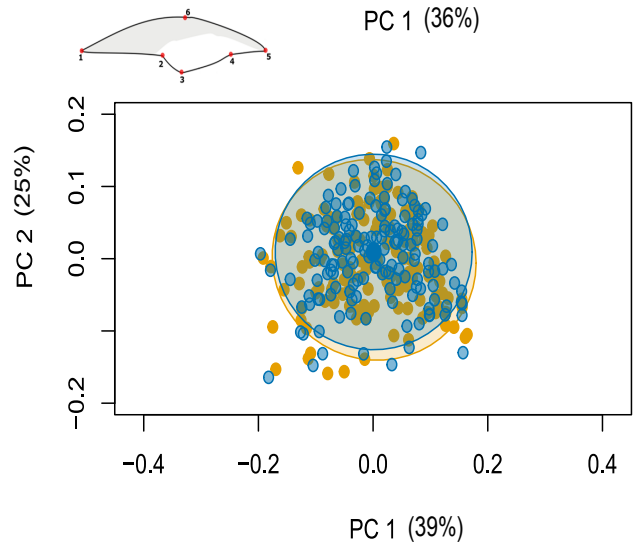

Supplement: S9 Appendix — Each dot represents an individual and the ellipses represent 95% CI for the distribution by replicates (large dot represents the mean for each morph replicates in these dimensions). (PDF) [file pone.0300359.s009.pdf]

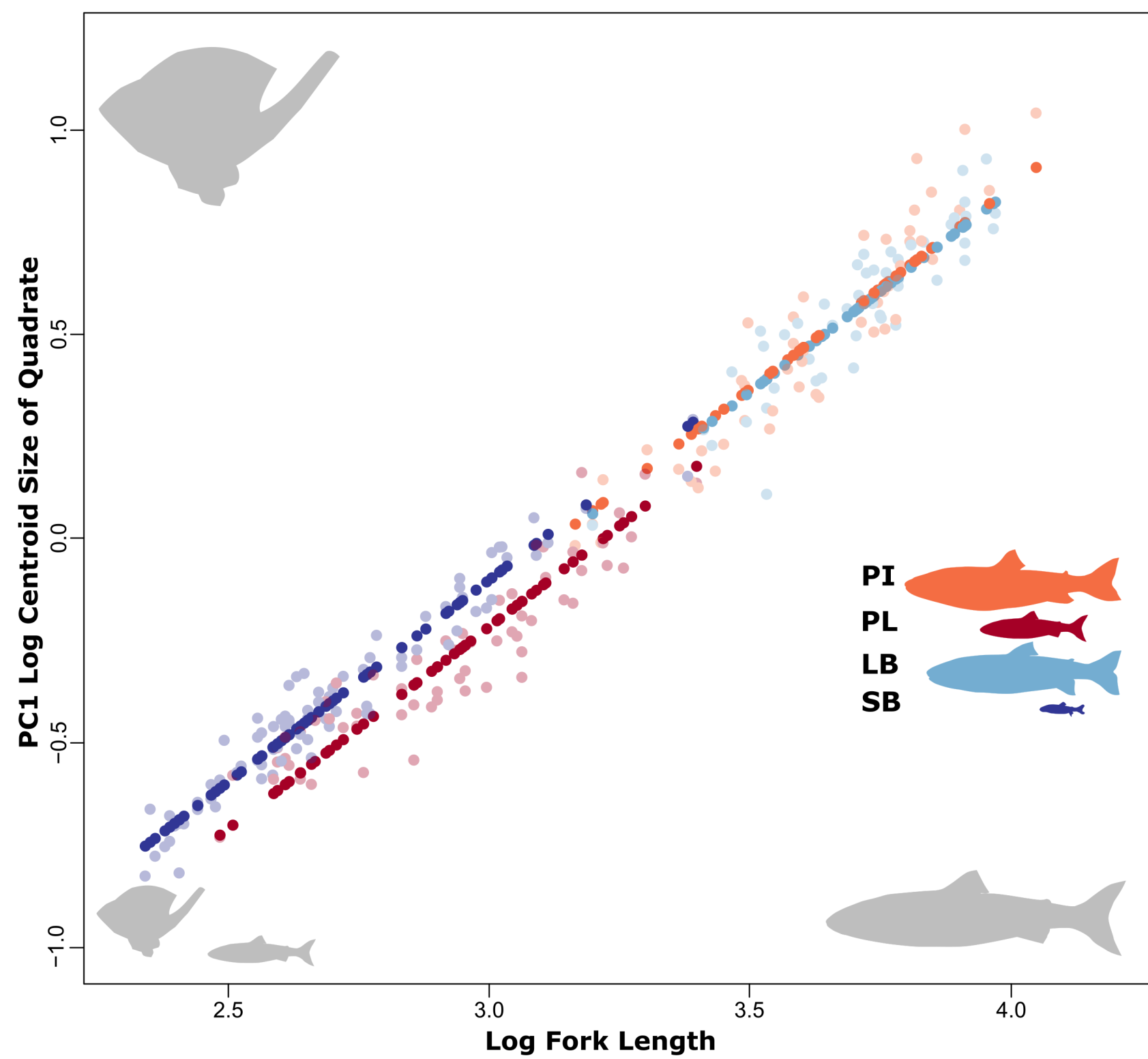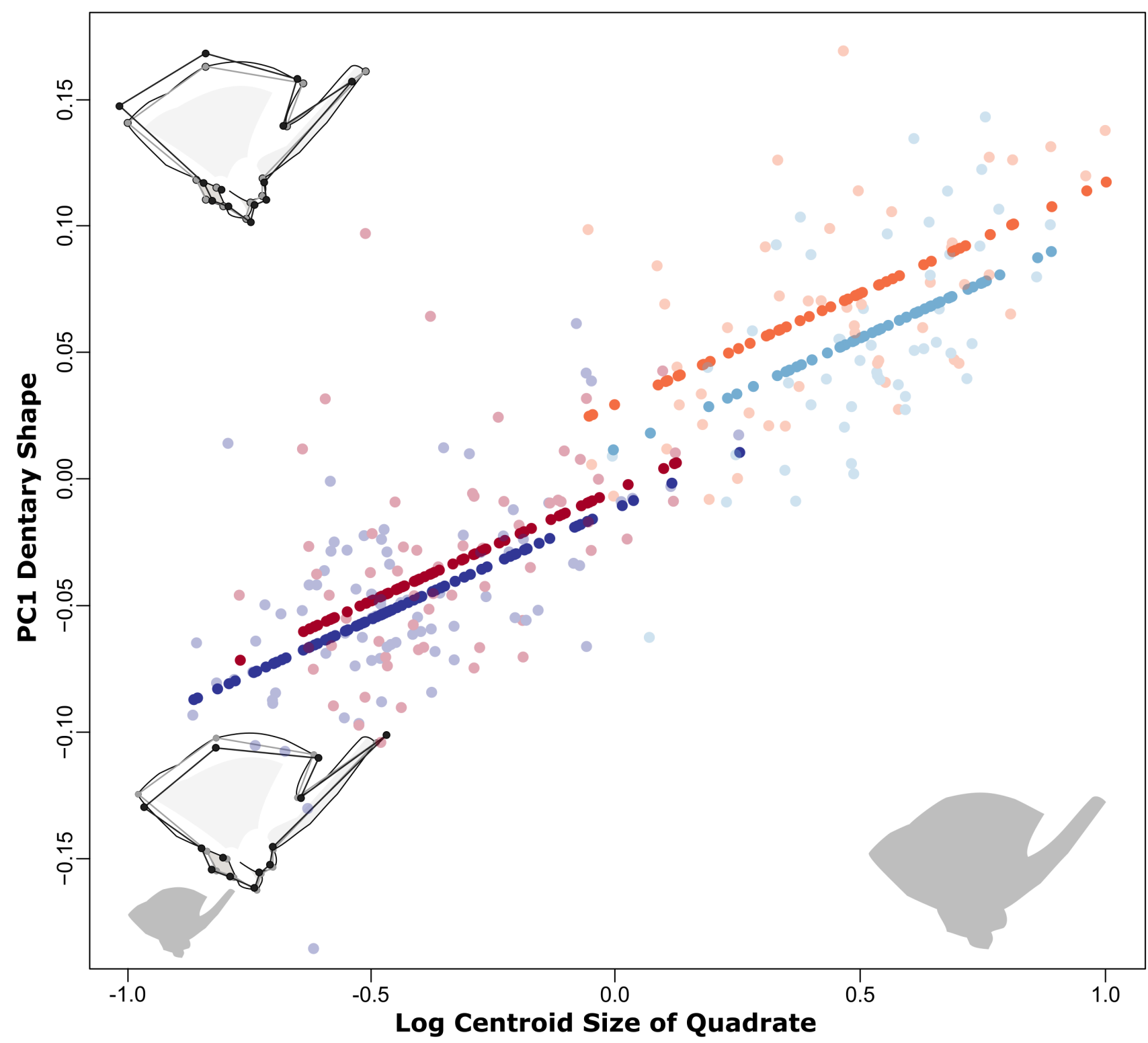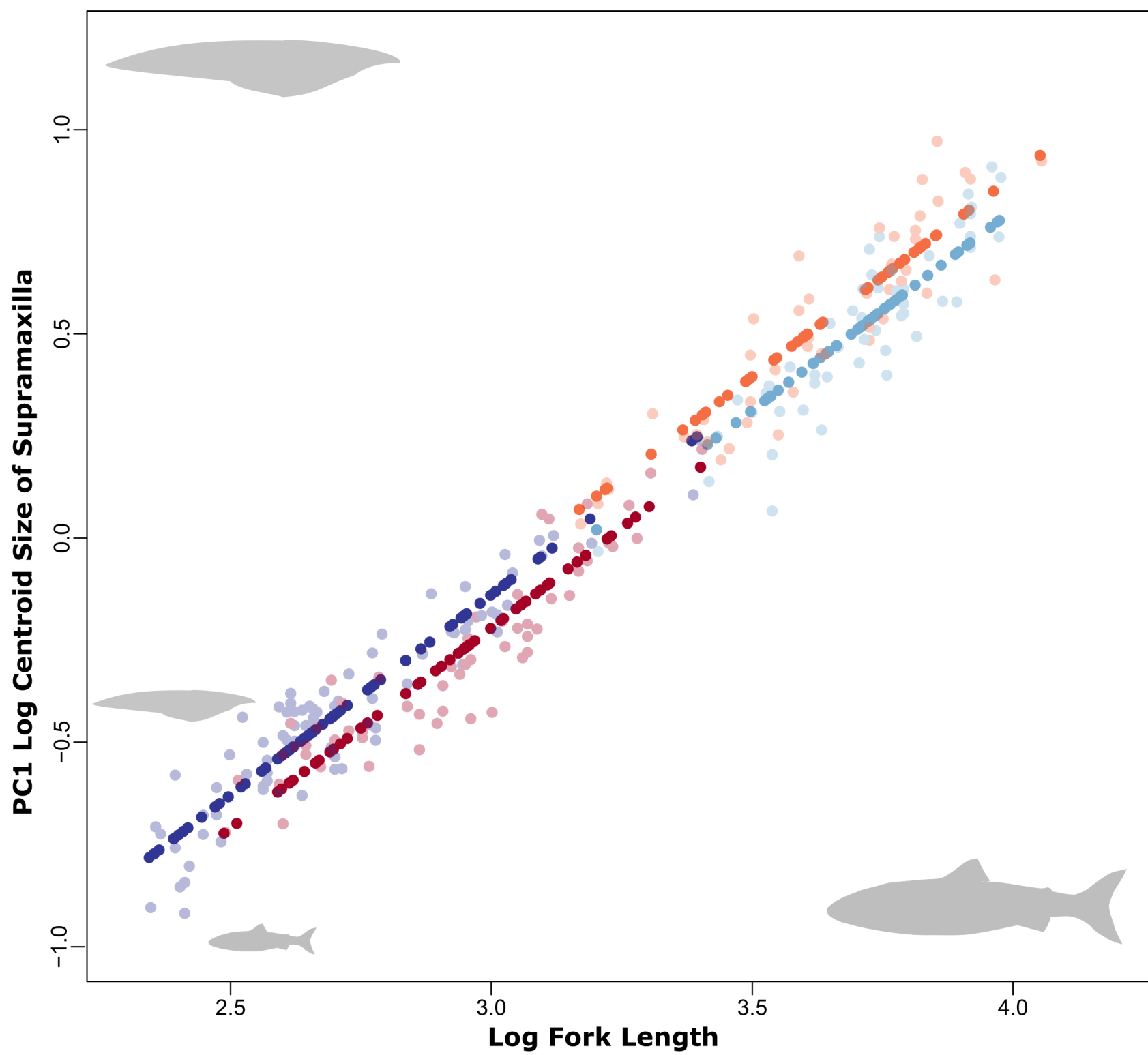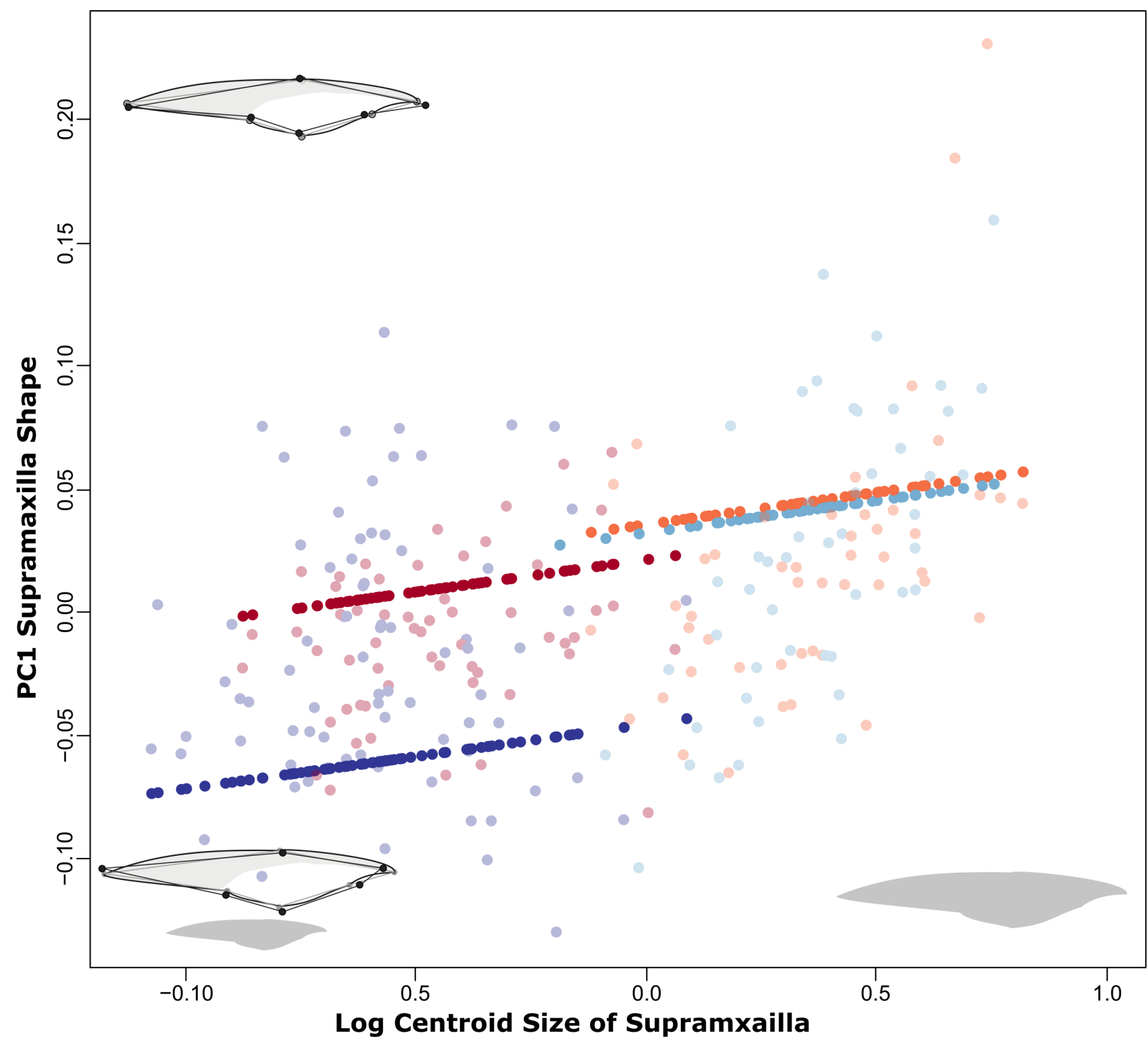

Supplement: S12 Appendix — Left panels: Relationships between bone size and fork length by morph; Right panels: Relationships between bone shape and bone size. On the right are inset the associated shape changes related to each component, grey outlines the mean shape, and black the extremes for each PC. Shown are values for individuals (open circles) and the predicted values (filled) of regressions for both bone size (CSbone) vs body size (loge FL) or bone-shape vs bone-size. Loge, natural log transformation. (PDF) [file pone.0300359.s012.pdf]

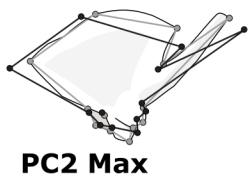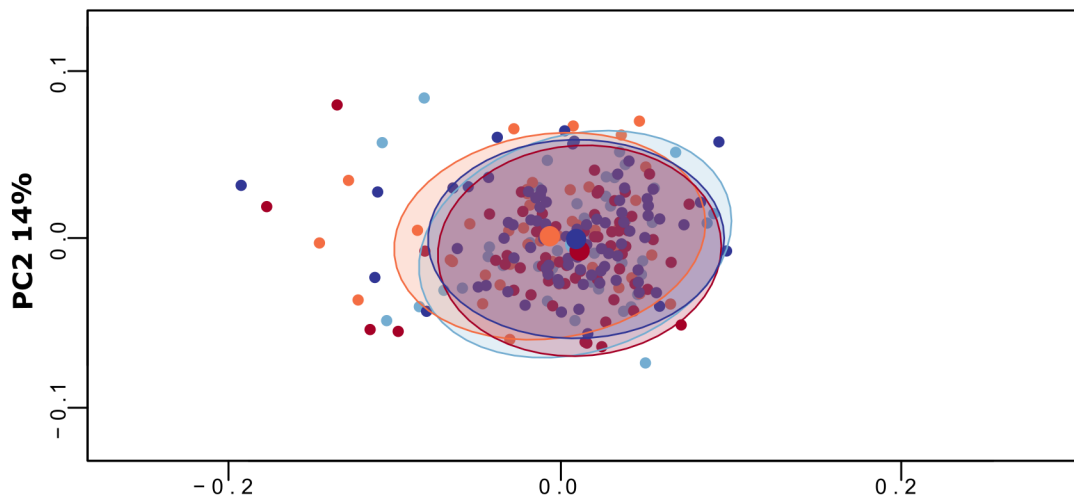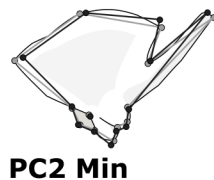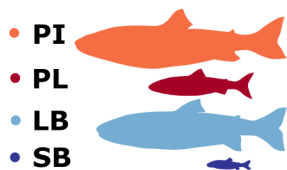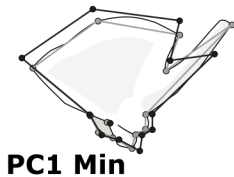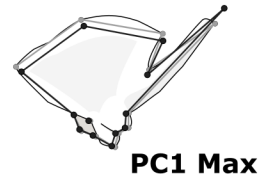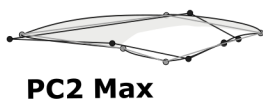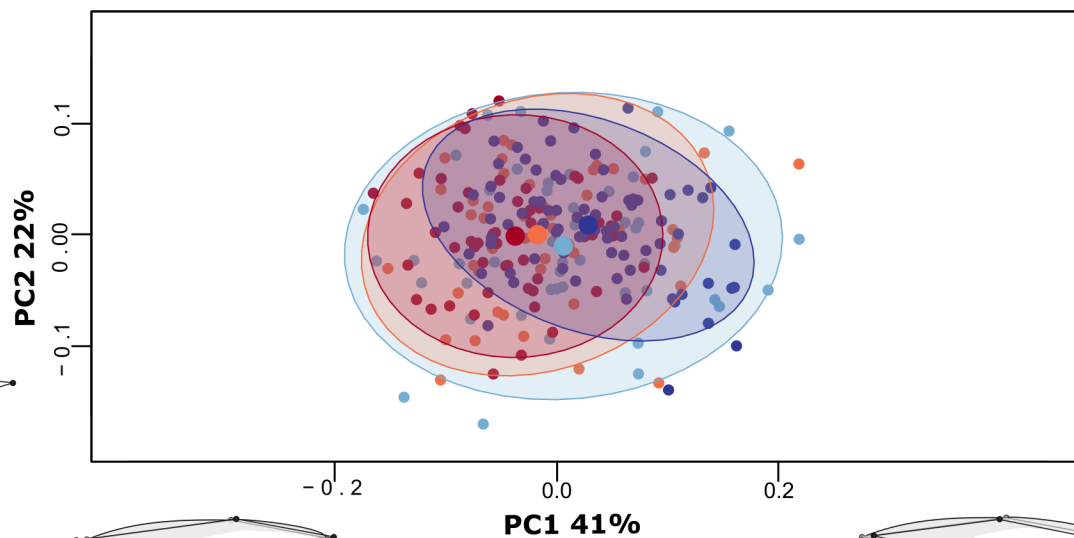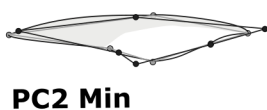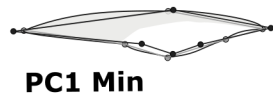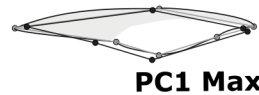

Supplement: S19 Appendix — On each axis are the associated shape changes related to each component, grey outlines the mean shape and black the extremes for each PC. Plots of shape warps on X- and Y-axis are unmagnified. Each dot represents an individual and the ellipses represent 95% CI for the distribution by morph (large dot represents the mean of each morph distribution in these two dimensions of shape). For the quadrate (top) PC1 and 2 explain 30% and 14% of the variation respectively and the supramaxilla (bottom) PC1 and 2 explain 40% and 22% respectively. (PDF) [file pone.0300359.s019.pdf]
