# Supplementary material for: Diversity in the internal functional feeding elements of sympatric morphs of Arctic charr (Salvelinus alpinus)
Source: PLoS One. 2024 May 21;19(5):e0300359. doi: 10.1371/journal.pone.0300359 (PMC11108142; doi:10.1371/journal.pone.0300359)
Supplement: S10 Appendix — Block 1 (x-axis) is replicate 1 and Block 2 (y-axis) is replicate 2. For all bones association between the replicates was always significant (p < 0.001). With the correlation coefficient being, dentary: 0.989, premaxilla: 0.939, articular-angular: 0.994, maxilla: 0.986, quadrate: 0.942 and supramaxilla: 0.835. (PDF) [file pone.0300359.s010.pdf]

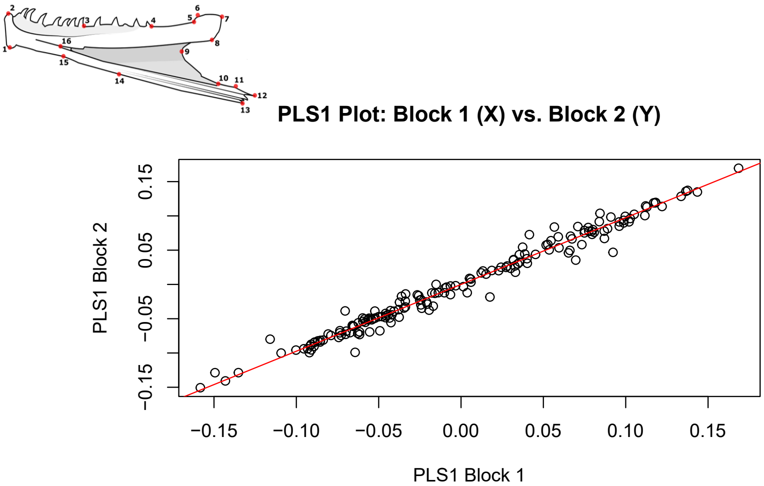
PLS1 Plot: Block 1 (X) vs. Block 2 (Y)
PLS1 Block 1
PLS1 Block 2

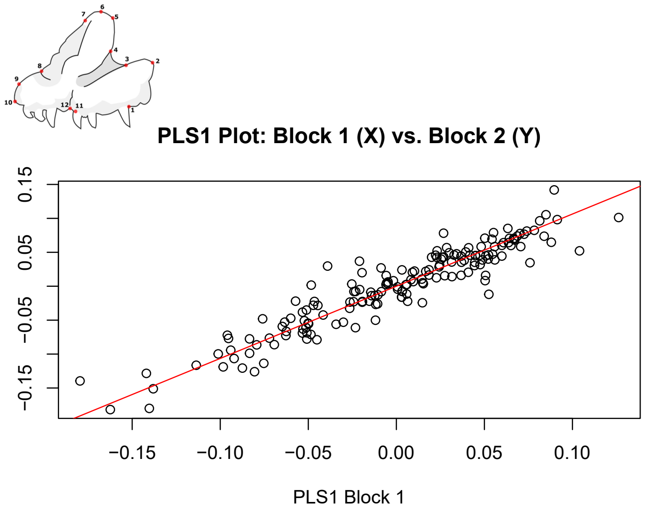
PLS1 Plot: Block 1 (X) vs. Block 2 (Y)
PLS1 Block 1
PLS1 Block 2

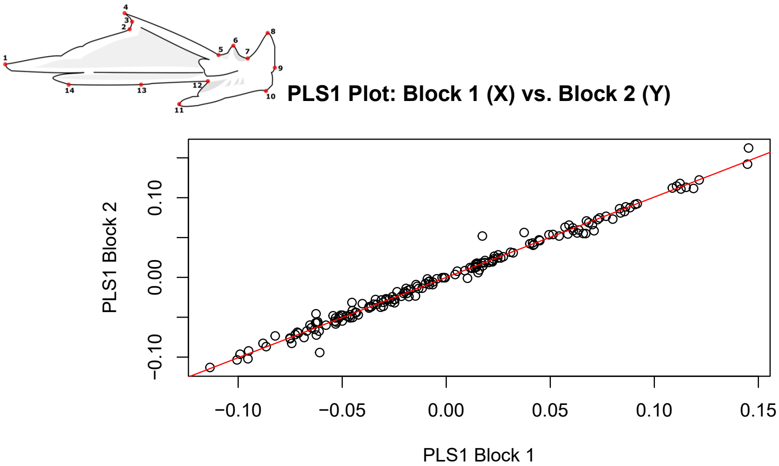
PLS1 Plot: Block 1 (X) vs. Block 2 (Y)
PLS1 Block 1
PLS1 Block 2

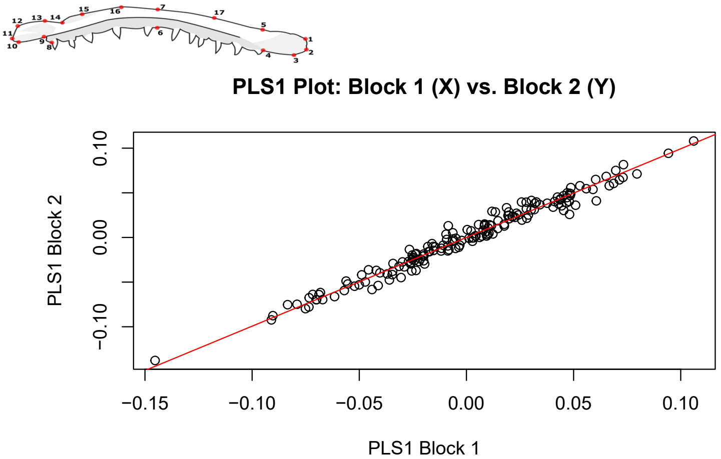
PLS1 Plot: Block 1 (X) vs. Block 2 (Y)
PLS1 Block 1
PLS1 Block 2

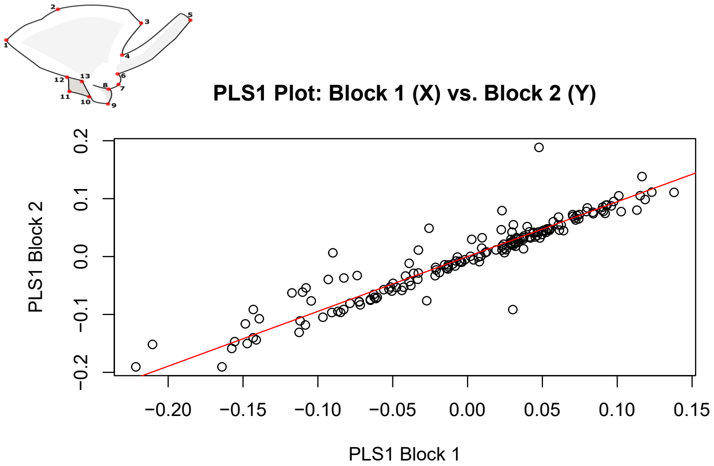
PLS1 Plot: Block 1 (X) vs. Block 2 (Y)
PLS1 Block 1
PLS1 Block 2

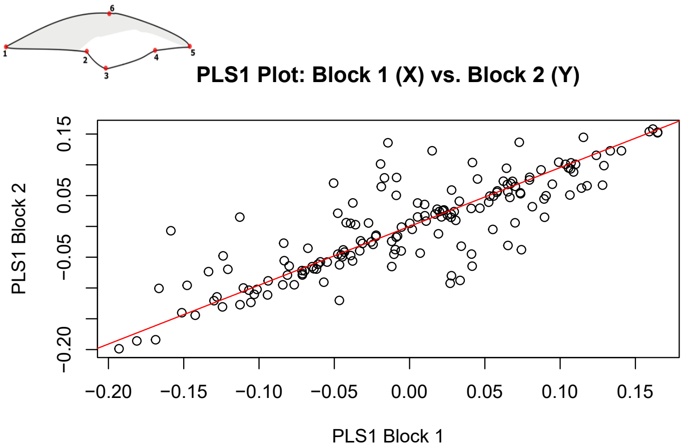
PLS1 Plot: Block 1 (X) vs. Block 2 (Y)
PLS1 Block 1
PLS1 Block 2
