# Supplementary material for: Diversity in the internal functional feeding elements of sympatric morphs of Arctic charr (Salvelinus alpinus)
Source: PLoS One. 2024 May 21;19(5):e0300359. doi: 10.1371/journal.pone.0300359 (PMC11108142; doi:10.1371/journal.pone.0300359)
Supplement: Attachment — Submitted filename: PONE-D-23-30771_review_MCG.pdf [file pone.0300359.s021.pdf]

Review for:

Diversity in the internal functional feeding elements of sympatric morphs of Arctic charr

Quick Summary:

The authors set out to investigate a few well-stated questions that included patterns of allometry in various craniofacial bones(1), bone shape across different ecomorphs(2), determine if morphologies differ along a determined bentho-pelagic axis(3), and test for patterns of integration between the upper and lower bones of the jaws across the different ecotypes(4). The authors laid out clear hypotheses and used methods that were exactly appropriate for testing.

1: The authors found allometric differences between the different ecomorphs, differences that included a sex x morph interaction. Differences were found to be across the whole-body and head shape.

2: The authors found significant allometric relationships across various craniofacial bones, many of which were different between PI x PL and PL x SB ecomorphs. The authors, appropriately, corrected for size on all visualizations.

3: Despite overlap in phenotypic space, the authors found supportive evidence of ecomorphs being partitioned into diverging regions, particularly the premaxilla, maxilla, dentary, and articular.

4: The authors found significant covariation between the upper and lower jaws, with magnitude of covariance being highest in dentary and articular – which could be expected. The authors found no significant differences in effect sizes between morphs of bone pairs.

Overall:

In truth, I looked over this manuscript and found nothing lacking. It is a straightforward, yet detail, geometric morphometric paper. The authors do diligent work and approach the system appropriate, with corrections and concerns in mind. With these studies, and with my own work, I typically look for corrections on size and how authors deal with this, the cleanliness and accuracy of figures, and the overall thoroughness of the discussion. I, personally, find the manuscript to be exceptional.

Specific Comments:

The figures are gorgeous. Extremely well done, easy to interpret, and quite clean in appearance.

Writing is clear and quite exceptional.

I quite appreciate the thoroughness of the discussion. Not only is it well written, but it is also expansive on covering ideas, theory, and bigger concepts.

Line Comments (for rolling line counts):

No specific line comments.
